# Dual-Network Thermal-Insulating and Flame-Retardant Cellulose Aerogel Fabricated via Ambient Pressure Drying

**DOI:** 10.3390/polym17172377

**Published:** 2025-08-31

**Authors:** Zhengsong Wu, Yucheng Gao, Shibin Nie, Dongyue Zhao, Xudong Cheng

**Affiliations:** 1 School of Safety Science and Engineering, Anhui University of Science and Technology, Huainan 232001, China; 2023200165@aust.edu.cn (Z.W.); zhaody2022049@aust.edu.cn (D.Z.); 2 School of Public Security and Emergency Management, Anhui University of Science and Technology, Hefei 231131, China; 3 State Key Laboratory of Fire Science, University of Science and Technology of China, Hefei 230027, China; chengxd@ustc.edu.cn

**Keywords:** dual-network, flame-retardant, cellulose aerogel, ambient pressure drying

## Abstract

Cellulose aerogel is a promising thermal insulation material with terrific thermal insulation and environmental friendliness. However, the intrinsic flammability of polysaccharide molecules and dependence on freeze-drying have limited its application in flame-retardant and thermal management systems. Here, we develop a flame-retardant biomass aerogel based on a dual-network matrix of bacterial cellulose and sodium alginate. This innovative material enables high-efficiency and low-cost preparation via ambient pressure drying technology (only ~3.5% volume shrinkage), while achieving flame retardancy by introducing an inorganic nanosheet microstructure within a polymer matrix. The resulting dual-network flame-retardant cellulose aerogel demonstrates thermal performance superior to that of most polymer foams and conventional cellulose aerogels, featuring an ultra-low thermal conductivity of ~0.04 W m^−1^ K^−1^ and a high limiting oxygen index (LOI) of ~69%. This research provides a novel strategy for simultaneous flame-retardant modification and energy-efficient manufacturing of biomass-derived aerogels.

## 1. Introduction

Benefiting from a unique porous microstructure, aerogel materials are endowed with lightweight properties and low thermal conductivity, demonstrating significant application potential in thermal management fields, including flame-retardant building materials, electronic devices, thermal protection, and aerospace applications [1,2,3,4]. However, traditional petrochemical polymer aerogels emit massive harmful gases or particles during manufacturing and utilizing process (e.g., phenolic aerogels release harmful formaldehyde), failing to meet the growing demand for environmentally friendly materials in thermal management applications [5,6]. As the most abundant natural polysaccharide on Earth [7], cellulose exhibits high availability and notable environmentally friendly and biodegradable advantages; thus, it can serve as an ideal alternative to non-renewable polymer [8,9,10,11,12,13]. Although cellulose aerogels exhibit terrific thermal insulation properties, the thermal cleavage of abundant hydroxyl groups within cellulose molecular chains causes inherent flammability and poor thermal stability of cellulose-derived aerogels, restricting their application in flame-retardant fields [14,15,16].

Guided by the principles of green chemistry and environmental sustainability, scientists have made considerable efforts to explore halogen-free flame-retardant modifications of cellulose. For instance, studies have reported the introduction of phosphorus or nitrogen elements onto the active sites of cellulose molecular chains through chemical grafting methods to promote char formation [17,18], thereby reducing heat release rates and enhancing flame retardancy. Alternatively, flame resistance has been achieved by physically blending inorganic hydroxides (such as magnesium hydroxide or aluminum hydroxide) [19,20]. Although these strategies improve flame retardancy, critical limitations persist in cellulose aerogel manufacturing: the abundant hydrogen bonds on anhydroglucose rings induce excessive water absorption and structural instability of cellulose, causing collapse during conventional drying processes due to liquid surface tension effects. As a result, drying of cellulose aerogels relies on freeze-drying solvent sublimation, which is inefficient and expensive [21,22,23,24]. Consequently, combining flame-retardant modification strategy with high-efficiency manufacturing techniques to realize efficient ambient pressure drying of flame-retardant cellulose-based aerogels remains a tremendous challenge [25,26].

Herein, we report a dual-network thermal-insulating and flame-retardant cellulose aerogel fabricated via high-efficiency ambient pressure drying strategy (APD). Initially, the strategy incorporates sodium alginate (SA) into bacterial cellulose (BC) as a flame-retardant component to enhance the fire resistance of biomass matrix. Subsequently, solvent replacement and cryogenic ionic cross-linking using glutaraldehyde (GLD) and metal ion-containing ethanol solutions (Ca^2+^) are performed. The coordination between Ca^2+^ ions and SA established a cross-linked network (Network I), while the aldehyde groups of GLD simultaneously reacted with hydroxyl groups abundant in both BC and SA, forming a secondary covalent network (Network II). This dual-network matrix can effectively reinforce cell wall strength, successfully counteracting capillary forces during solvent evaporation and enabling ambient pressure drying with minimal volumetric shrinkage (~3.5%). On a dual-network basis, introducing inorganic montmorillonite nanosheets to create flame-retardant lamellar microstructure synergistically enhances mechanical robustness and fire resistance (minimal thermal conductivity: 0.043 W m^−1^ K^−1^; UL-94 V-0 rating). This work proposes a groundbreaking strategy to realize flame-retardant modification and high-efficiency ambient pressure drying of cellulose aerogels simultaneously through synergistic integration of dual-network component design and inorganic nanosheet structure.

## 2. Materials and Methods

### 2.1. Chemicals

All reagents and solvents were of analytical quality and used as received. Bacterial cellulose solution (BC, 0.8 wt.%) was purchased from Guilin Qihong Technology Co., Ltd. from Guilin (China). Sodium alginate (SA, 90%, M/G ratio = 2:1) was purchased from Macklin Biochemical Co., Ltd. from Shanghai (China). Glutaraldehyde (GLD, 50%, photographic grade) and montmorillonite (MMT, K-10) were acquired from Shanghai Aladdin Biochemical Technology Co., Ltd. from Shanghai (China). Deionized water was self-made in the laboratory. Characterization of nanomaterials (MMT nanosheets and BC nanofibers) is shown in Figures S1–S3. All other reagents were used without further purification.

### 2.2. Instrumentation

Fourier-transform infrared (FTIR) characterization was conducted with an infrared spectrometer (Nicolet 380, ThermoFisher Scientific, Waltham, MA, USA) based on potassium bromide (KBr) powder compression method: a small amount of sample is evenly mixed with KBr powder and thoroughly ground in an agate mortar. The mixture is then pressed into transparent, uniform thin sheet using a pellet press for FTIR testing (wavenumber range: 400 to 4000 cm^−1^). Scanning electron microscopy (SEM) was performed with a scanning electron microscope (G450, Zeiss, Oberkochen, Germany) at the acceleration voltage of 2 kV in SE2 mode. Each sample was subjected to a 100 s gold sputtering treatment (30 mA of constant current) before observation. Transmission electron microscopy (TEM) was conducted using a transmission electron microscope (JEM 2100 PLUS, JEOL, Tokyo, Japan) at the acceleration voltage of 200 kV. The thermal infrared images were captured using an infrared camera (VarioCAM^®^ hr head 680, InfraTec, Dresden, Germany). Thermal conductivity was measured by a Hotdisk 2500 s (Hotdisk, Shanghai, China) using transient plane source; two identical pieces of one sample were in contact with the thermal detector. Limiting oxygen index (LOI) and vertical burn rate (UL-94) tests followed GB/T 2406.2-2009 and GB/T 8333-2022 standards [27], where the sample size used in LOI test is 100 mm × 6.5 mm × 3 mm, and size used in UL-94 test is 130 mm × 13 mm × 3 mm. Heat release rate test (HRR) was carried out with a Cone Calorimeter (FTT007, FTT, Glasgow, UK), following ISO-5660-1 standard (heat flux: 50 kW/m^2^) [27]. Thermogravimetric analysis (TGA) was performed by a TGA/SDTA 851 e thermal analyzer (Mettler-Toledo, Zurich, Switzerland), heating from room temperature to 600 °C at 10 °C/min (air atmosphere). X-ray photoelectron spectroscopy (XPS) was conducted by ESCALAB Xi^+^ (ThermoFisher Scientific, Waltham, MA, USA). Raman scattering spectra were obtained with a System 2000 spectrometer (Renishaw, Gloucestershire, UK). All performance data reported are the average values of at least three parallel samples. Each parallel sample was independently prepared, processed, and tested to ensure the reliability and reproducibility of the results.

### 2.3. Preparation of MMT Nanosheets

MMT nanosheets were exfoliated according to the previously reported method [28]. Specifically, 5 g of MMT powder was added to 1000 mL deionized water suspension (0.5 wt.%) and vigorously stirred for one week, followed by 2 h ultrasonic dispersion to ensure sufficient delamination. The suspension was allowed to settle for 5 min, and the supernatant was then collected and centrifuged at 3000 rpm, followed by vacuum drying at 60 °C to obtain the MMT nanosheets.

### 2.4. Preparation of BS and BSM-X Aerogels

**Figure 1:**
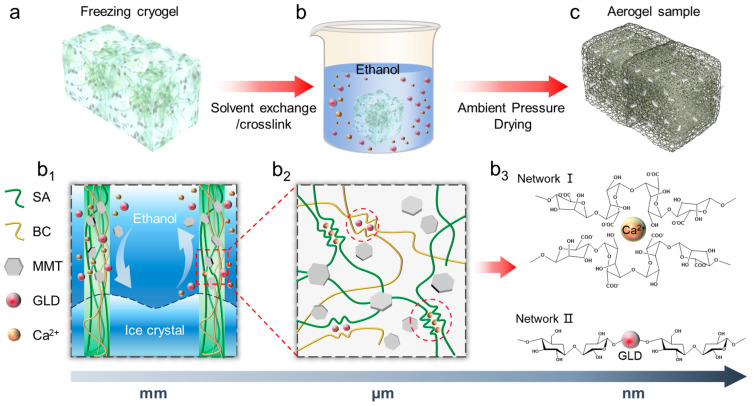
Schematic illustration showing the ambient pressure drying (APD) process and dual-network cross-linking mechanism of BSM aerogel. (**a**) Freeze-casting of slurry which consists of deionized water, SA, and BC. (**b**) Immerging the cryogel in ethanol solution which consists of Ca^2+^ and GLD at subzero temperature (e.g., −18 °C) for (**b_1_**) synchronous solvent exchange and ionic cross-linking of polymer binders along with the ethanol–ice interface pushing forward. (**b_2_**) Once the ice is dissolved, the Ca^2+^ and GLD diffuse inside the walls and cross-link the SA and BC immediately. (**b_3_**) Formation of the “egg-box” complexation between G blocks on SA chains and Ca^2+^. Once the ice is dissolved, the Ca^2+^ diffuses inside cell walls and cross-links the SA immediately, forming the “egg-box” complexation, and GLD simultaneously cross-links with BC to form a cross-linked network. (**c**) After the thorough solvent exchange, aerogel can be dried by APD strategy.

The fabrication process of aerogels is illustrated in Figure 1a. Initially, 0.8 wt.% BC solution was thoroughly mixed with 2 wt.% SA solution under stirring for 30 min (1:1 volume ratio). The homogeneous mixed precursor solution was then transferred into a pre-cooled mold and subjected to freezing at −18 °C for 3 h to obtain completely solidified frozen cryogel. Simultaneously, a cross-linking solution was prepared by dissolving calcium chloride (CaCl_2_ 0.05 mol/L) in 500 mL ethanol, followed by adding GLD at a volume ratio of 1:200 (glutaraldehyde:ethanol), which was subsequently stored under refrigeration (−18 °C). The cryogel was immersed in the cross-linking solution and maintained at −18 °C for 36 h to complete the cross-linking process. Finally, the thoroughly soaked sample was dried in an oven at 60 °C under ambient pressure for 1 h to obtain composite aerogel (the aerogels without MMT nanosheets are marked as BS). Composite aerogel with MMT nanosheets was fabricated via a similar process, by adding different contents of MMT nanosheets to precursor solution. The obtained aerogel with MMT is named BSM-X, where X means the weight ratio of MMT nanosheets content. For instance, BSM-1 means 1 wt.% of MMT nanosheets in the mixture solution of BC and SA.

### 2.5. Mechanical Test of BS and BSM-X Aerogels

Static mechanical compression and dynamic fatigue resistance tests were conducted using a 5565A (Instron, Boston, MA, USA) with a 5000-N loading cell; each sample was cut and polished to a standard size of a cube with a side length of 10 mm, and at least three samples were tested for each component ratio (2 mm/min of strain ramp rate). The apparent density of each sample was calculated by the ratio of sample mass to its apparent volume. The volume shrinkage ratio was obtained by the ratio of volume before drying to volume after drying.

### 2.6. Cost Analysis of Ambient Pressure Drying

To compare the two strategies, APD and FD, a rough cost estimation was conducted. We compared the total time cost and financial cost required to dry 1 m^3^ of samples for both methods (for each batch, the sample volume occupies 50% of the freeze dryer chamber or freezer) (Tables S1 and S2).

1. FD costs: to prepare large-sized samples as much as possible and improve the preparation efficiency, a large freeze dryer (FreeZone^®^ 18L Freeze Dryer Labconco^®^, Kansas City, MO, USA) with a chamber volume of 18 L was used for freeze-drying (¥266,000). FD method requires dividing the drying of 1 m^3^ of samples into 112 batches, with each batch set to a freeze-drying time of 24 h, resulting in a total time consumption of 2688 h (power consumption was 2 kW/h). Therefore, the required electricity cost was CNY 3225 (2 kW × 2688 h × ¥CNY 0.6 = CNY 3225). The vacuum pump oil (Edward 19# pump oil: ≈CNY 300/L) was changed every 30 days, so the material cost incurred was CNY 1120 (112 batches/30 days/time × CNY 300 = CNY 1120).

2. APD costs: the price of a common refrigerator (with a capacity of 210 L) is approximately CNY 4000. The refrigerator processes 105 L of samples each time, requiring only 10 batches, with each batch set to a solvent exchange time of 36 h, resulting in a total time consumption of 360 h (power consumption is 0.5 kW/h). Therefore, the required electricity cost is CNY 108 (0.5 kW × 360 h × CNY 0.6 = CNY 108). Each batch of samples consumes 10 times its volume of ethanol (1050 L; unit price, CNY 3.6/kg) for solvent exchange (CaCl_2_, 0.05 mol/L; unit price, CNY 2.5/kg), and 0.5% volume fraction of GLD (unit price ¥15/kg) needs to be added to the ethanol. Therefore, the material cost is ~CNY 3785 (total ethanol: 1050 L × 0.789 kg/L × CNY 3.6 = CNY 2982; total CaCl_2_ cost: 1050 L × 0.05 mol/L × 0.111 kg/mol × CNY 2.5 = CNY 14.6; total GLD cost: 1050 L × 0.5% × CNY 15 = CNY 788).

In conclusion, APD method saves CNY 100 for fabrication of each 1 m^3^ sample compared to FD method ((CNY 3225 + CNY 1120) − (CNY 108 + CNY 3785) = CNY 452), and the production time is reduced by 87%, significantly lowering financial and time cost and demonstrating higher potential for scalable production.

## 3. Results and Discussion

### 3.1. Material Synthesis and Characterization

**Figure 2:**
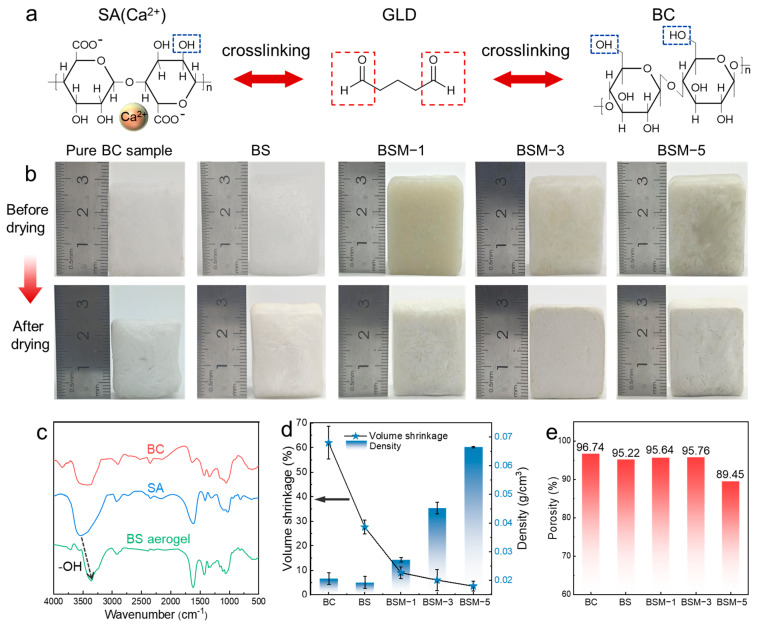
BS and BSM aerogels fabricated by APD strategy. (**a**) Acetalization reaction cross-linking mechanism of BC/SA dual network. (**b**) Optical photographs of ethanol-soaked BC, BS, and BSM gels with various MMT contents before drying and aerogels after drying. (**c**) FT-IR spectra curve of BC, SA, and BS aerogel. (**d**) Apparent density and volumetric shrinkage change curves of pure BC, BS, and BSM aerogels. (**e**) Porosity of pure BC, BS, and BSM aerogels.

The cellular microstructure of dual-network cellulose aerogel is constructed via the freeze-casting strategy. During the ice crystal nucleation process, BC nanofibers and SA molecules are compressed by ice crystals to form a uniform cell wall structure. The fully frozen cryogel is then soaked in a subzero temperature ethanol bath (−18 °C) for solvent exchange, during which ice crystals in the sample are gradually etched by the mutual solubility of ethanol and water (Figure 1(b_1_)). As the solid–liquid interface continues to advance, the composite pore walls of BC and SA are fully exposed to the ethanol solution of metal ions (Ca^2+^) and GLD, where the metal ions undergo an “egg-box” cross-linking reaction with the G-blocks of SA, leading to the formation of a gelled molecular network (Network I) [29]. This cross-linking reaction can be proofed by Fourier-transform infrared (FTIR) curves which demonstrate a red shift of asymmetric vibration expansion in the peak of the carboxyl group (~1610 cm^−1^) (Figure 1(b_3_) and Figure S4). In addition, the aldehyde groups of GLD undergo acetal cross-linking with the abundant hydroxyl groups on SA and BC simultaneously, forming Network II (Figure 2a) [30]. FTIR curves further demonstrate the cross-linking process of the dual network: BC exhibited characteristic cellulose structures with a broad peak at 3350–3550 cm^−1^ attributed to -OH groups [8,30]. Compared to SA, the BS curve displays a similar IR spectrum but with a sharper -OH peak at 3360 cm^−1^. Notably, the broad -OH peak observed in BC and SA disappear, and red shift appears in the BS curve, indicating a significant reduction in -OH groups [30]. This suggests acetal cross-linking of both SA and BC via reactions between -CHO groups of GLD and -OH groups of BC/SA (Figure S5) [30].

Benefiting from the BC/SA dual-network cross-linked structure, constructed through the above subzero temperature solvent exchange and synergistic interaction of Ca^2+^ ions and GLD, the mechanical strength of cell walls is significantly enhanced [31]. This allows the microstructure of the BS aerogel to resist the capillary shrinkage caused by solvent evaporation during the subsequent APD process, ensuring structural integrity and a lower volume shrinkage rate after ambient drying. As Table 1 shows, a clear trend can be seen: as MMT is added, the volume shrinkage rate decreases significantly, and the higher the MMT content, the lower the volume shrinkage rate. Pure BC aerogel prepared via freeze and ambient pressure drying exhibited an extremely high volume shrinkage rate of 62.01%, indicating that pure bacterial cellulose struggles to produce usable aerogels under ambient pressure conditions. However, after dual-network cross-linking, the resulting BS aerogel demonstrated a significantly reduced volume shrinkage rate of 27.63%, marking a 34.38% decrease compared to pure BC aerogel (Table 1, Figure 2b,d). Furthermore, with the addition of MMT nanosheets, MMTs construct an inorganic nanosheet layer structure within polymer networks, further enhancing the strength of cell walls; the resulting BSM aerogel exhibited progressively lower volume shrinkage rates. Notably, as MMT content increases, the volume shrinkage rate of BSM-5 drops to a negligible 3.54% (Figure 2d).

In addition, all the dual-network BS and BSM aerogels prepared by the APD method exhibit extremely high porosity (Figure 2e). High porosity is a critical indicator for evaluating the successful preparation of aerogels, indicating the integrity of the microstructure (with no significant structural shrinkage or collapse) and further confirming the universality of the dual-network system for the APD method. Interestingly, as the main component of the antibacterial agent, GLD not only acts as cross-linking agent but also significantly inhibits the decomposition of BS aerogels under microbial action, thereby enhancing their durability (Figure S6) [32]. Moreover, compared to freeze-drying technology that requires a high vacuum-sealed environment, the dual-network aerogels prepared using the APD technique achieved an 87% increase in drying efficiency while ensuring the integrity of the microporous structure along with significantly reduced financial and time costs, demonstrating great potential for scaling-up and continuous production (Figure S7 and Tables S1 and S2).

### 3.2. Structural and Mechanical Properties’ Characterization

**Figure 3:**
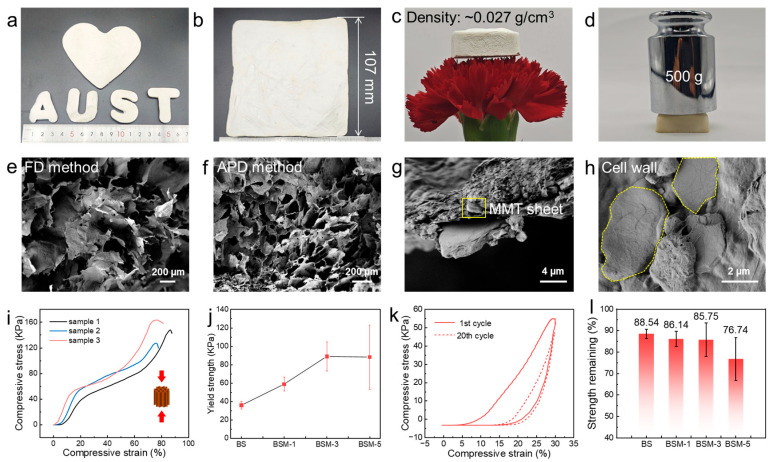
Microstructure and mechanical performance of BSM aerogel. (**a**) BSM samples with various shapes. (**b**) Large-size BSM sample fabricated by APD methods. (**c**,**d**) Optical photographs of BSM aerogel demonstrating advantages of the material’s light weight and high strength: BSM aerogel with a density of 0.027 g cm^−3^ standing on fresh petals (**c**); BSM aerogel can withstand the pressure of a 500 g weight (**d**). (**e**,**f**) SEM image of cellular microstructure of BSM-1 aerogels fabricated by FD method (**e**) and APD method (**f**). (**g**,**h**) SEM images of the BSM cell wall cross-section (**g**) and pore wall surface (**h**) demonstrating that MMT nanosheets are firmly anchored within BC/SA dual network. (**i**) σ–ε curves of BSM-1 sample in compression test. (**j**) Yield strength of BS and BSM aerogel with various MMT contents. (**k**) σ–ε curves of BSM-1 sample during 20 cycles of compressing fatigue resistance test (ε = 30%). (**l**) Strength remaining of BS and BSM aerogel with various MMT contents.

Due to the removal of restrictions imposed by the closed drying space of freeze-drying, the dual-network BSM aerogels with varied shapes can be fabricated to satisfy diverse application scenarios by using different modes, and they can be conveniently produced in larger sizes (100 mm × 100 mm × 15 mm) by the APD method, fully demonstrating the advantages of the dual-network system in terms of processability and scalability (Figure 3a,b). The mechanical reinforcement of cell walls provided by the BC/SA dual-network matrix makes sure that BSM aerogels can overcome the capillary forces during atmospheric pressure drying, ensuring the integrity of both the microstructure and macroscopic samples. The BSM samples dried by the APD method exhibit a homogeneous cellular microstructure similar to that of samples dried by the FD method (Figure 3e,f). The uniform and dense cell wall morphology indicates good interface compatibility between the MMT reinforcements and the polymer matrix, which is considered to be the key structural basis for the subsequent synergistic improvement of thermal insulation, flame retardancy, and mechanical stability.

Furthermore, the MMT nanosheets are firmly bonded to cell walls by BC nanofibers (Figure 3g,h), which can provide stress transfer and support to cell walls under compressing load. This allows the compressive strength of BSM aerogels to withstand stress loads equivalent to ~2000 times their own weight, providing the aerogel its lightweight and high-strength characteristics (with the addition amount being only 1–5 wt.%) (Figure 3c,d). Taking the σ–ε curve of BSM-1 as an example, the curve shows a discernible yield point at the strain of ~10%. This is due to the elastic buckling and wrinkling of cell walls under load, with a yield strength of ~60 KPa, which is higher than some reported biomass porous materials like cellulose foams, aramid nanofiber aerogels, carbon nanotube aerogels, and other porous materials [33,34,35,36]. As compressive strain increases, cellular structure enters the compression densification stage, leading to an increase in compressive stress; the maximum stress is increased to 378.9 KPa (BSM-3, ε = 84%) (Figure 3i and Figure S8). With the increase in the content of MMT nanosheets, the compressive strength of BSM aerogels gradually improves, reaching a maximum when the MMT content is 3 wt.% (BSM-3). Compared to BS samples without MMTs, the strength of BSM increases by ~150%. It is speculated that a continuous inorganic sheet structure is constructed on cell walls, enabling effective load transfer and reinforcement of the wall structure. The strengths of BSM-3 and BSM-5 are similar, which is attributed to the excessive amount of MMTs not being sufficiently covered by BC nanofibers to achieve an effective mechanical enhancement effect (Figure 3j). Notably, BSM aerogels also exhibit a certain flexibility, maintaining over 75% of maximum stress retention after 20 loading–unloading cycles (ε = 30%), which demonstrates considerable fatigue resistance and mechanical stability for long-term application conditions (Figure 3k,l and Figure S9).

### 3.3. Thermal Insulation Performance

**Figure 4:**
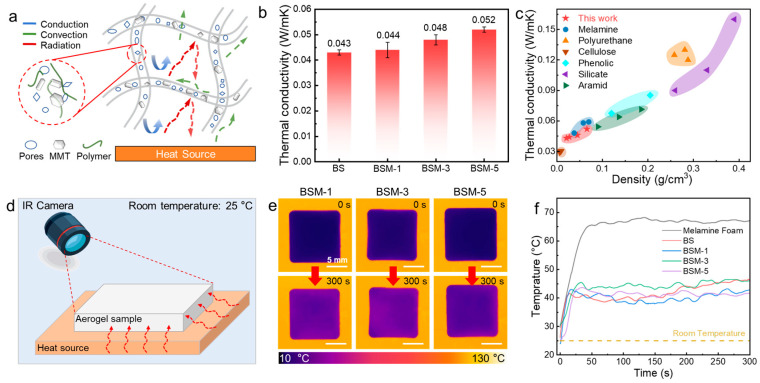
Thermal insulation characterization of BS and BSM aerogels. (**a**) Schematic illustration of aerogel showing various internal thermal insulation mechanisms. (**b**) Thermal conductivities of BS and BSM samples. (**c**) Ashby plotting of thermal conductivity–density for BS and BSM aerogels and other reported polymeric and ceramic porous materials. (**d**) Schematic of visual thermal insulation test recorded by an infrared (IR) camera. (**e**) IR photographs of BSM aerogels recorded with for 300 s which show surface temperature change of each sample during test. (**f**) Temperature–time curves of different samples in 300 s heating process.

BS and BSM aerogels are endowed with extremely high porosity (ranging from 89% to 95%) and very low density (ranging from 0.14 g cm^−3^ to 0.64 g cm^−3^). Such a porous honeycomb-like microstructure can effectively reduce heat transfer (including convection, conduction, and radiation). For aerogel materials, thermal convection is the primary factor affecting insulation performance; cell wall structure hinders the convection of air within the aerogel, extending the heat transfer path. Additionally, the multilayer cell wall structure creates multiple gas–solid interfaces, greatly enhancing phonon scattering and multilayer reflection effects, thereby reducing thermal radiation and significantly improving thermal resistance efficiency (Figure 4a). As a result, both BS and BSM aerogels exhibit extremely low thermal conductivity λ (0.043–0.052 W m^−1^ K^−1^) (Figure 4b), which is relatively close to that of pure cellulose aerogels prepared by freeze-drying and significantly lower than that of many reported polymeric and ceramic porous materials (such as melamine sponge, phenolic foam, and porous silicate cement, etc. [37,38,39,40,41,42]) (Figure 4c).

To visually demonstrate the thermal insulation performance brought about by the low thermal conductivity, a visual thermal insulation test was conducted on BS and BSM aerogel samples (25 mm × 25 mm × 10 mm), as well as on commercial melamine insulation foam. Five samples were placed on a constant temperature heating platform at 130 °C for a heating process (lasting 300 s, room temperature of 25 °C), during which the surface temperature changes were recorded by a thermal infrared camera to assess their thermal insulation performance (Figure 4d). Within 49 s, the temperature of commercial foam rapidly increased to 65.6 °C and stabilized at 65.7 °C. In contrast, the BS aerogel temperature only raised to 46.2 °C, eventually stabilizing at 48.5 °C. Additionally, the addition of inorganic MMTs do not lead to a significant decrease in thermal insulation performance: BMS-1 stabilized at a final temperature of 46.1 °C, BSM-3 stabilized at 48.5 °C, and BSM-5 stabilized at 41.5 °C, demonstrating a temperature difference of over 80 °C compared to the exposed heating platform surface. This indicates excellent thermal insulation performance, with surface temperatures ~25 °C lower than that of commercial insulation foam (Figure 4e,f and Figure S10). Based on the thermal conductivity of BS and BSM, as well as the final steady-state surface temperature measured by the infrared camera, the heat flux Q can be derived using the heat flow calculation formula ($Q=\frac{\lambda \cdot A\cdot (\Delta T)}{d}$; Q is heat flux (W), λ is thermal conductivity (W m^−1^ K^−1^), ∆T is the temperature difference between upper and lower surfaces (K), A is the area of sample (m^2^), and d is the thickness of sample (m)). According to the calculation, the heat flux density of BS and BSM aerogels range from 350.4 to 460.2 W/m^2^ (Figure S11). Using BSM-1 as an example (heat flux density of BSM-1 is 369.2 W/m^2^), it represents a ~65% reduction in heat loss compared to the exposed surface (1050 W/m^2^), demonstrating excellent thermal insulation performance.

### 3.4. Thermal Stability and Flame Retardancy

**Figure 5:**
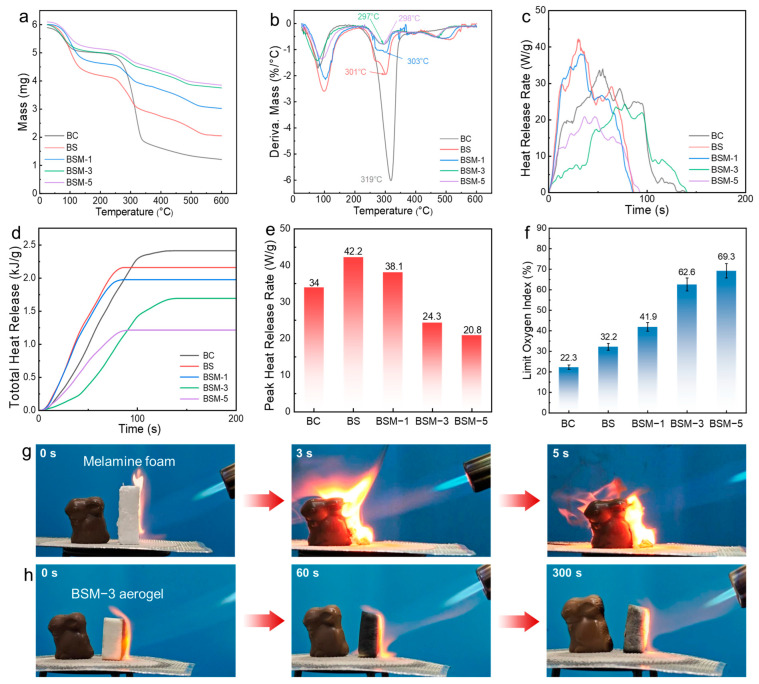
Thermal stability and flame retardancy of BS and BSM aerogels. (**a**) TGA curves of BS and BSM aerogels. (**b**) DTG curves of BS and BSM aerogels. (**c**) Heat release rates of BS and BSM aerogels. (**d**) Total heat release rates of BS and BSM aerogels. (**e**) Peak heat release rates of BS and BSM aerogels. (**f**) Limiting oxygen index of BS and BSM aerogels. (**g**,**h**) Comparison of commercial melamine foam and BSM-3 in butane flame scorch.

Thermal stability determines the reliability and application potential of aerogel materials in extreme thermal management scenarios. The thermal stability of pure BC, BS, and BSM aerogels was evaluated through thermogravimetric analysis (TGA) under an air atmosphere. Each sample was heated from room temperature to 600 °C at a rate of 10 °C/min. As shown in the TG and DTG curves (Figure 5a,b and Table 2), all samples exhibited similar TGA behaviors, with two distinct peaks in the DTG curves. The first peak in the range of 25~110 °C corresponded to moisture evaporation, while the main decomposition stage occurred in the range of 250~400 °C. Pure BC showed a maximum decomposition rate of 6.00%/°C at 319 °C (T_max%_), attributed to cellulose chain scission and volatile release. Thermal degradation under oxidative conditions involved BC macromolecular breakdown and subsequent char layer oxidation. Pure BC demonstrated a 5% weight loss temperature (T_5%_) of 69.83 °C and T_max%_ of 319 °C, with a low char yield of 9.09 wt.%. Compared to BC, BS and BSM composites exhibited higher initial decomposition temperatures, slightly lower T_max%_, but significantly enhanced char residues. Notably, BSM-5 achieved a char yield of 59.60%, representing a 50.51% improvement over BC. This enhancement arises from MMT’s barrier effect restricting volatile release, coupled with catalytic carbonization mediated by Al^3+^ in MMT and Ca^2+^ in the BC/SA dual network, which synergistically promoted the formation of high-quality protective char layers [43], thereby substantially improving the aerogels’ thermal stability [44].

To further analyze the thermal stability and thermal decomposition behavior of BS and BSM aerogels under extreme high-temperature conditions, microscale combustion calorimetry (MCC) testing was used as another method for studying the heat release characteristics, effectively simulating their exothermic behavior during thermal decomposition (Figure 5c–e). Pure BC aerogel reached its maximum heat release rate (HRR) of 33.99 W/g at 30 s, with a total heat release (THR) of 2.41 kJ/g within 150 s. Notably, after cross-linking with SA and Ca^2+^, the aerogel’s peak HRR increased to 42.23 W/g, while the THR decreased by 12.86%. Furthermore, no significant heat release was observed after 100 s. With the additional incorporation of MMTs (1%, 3%, and 5%), both the HRR and THR of the composite materials showed significant reductions. Specifically, the peak HRR values for BSM-1, BSM-3, and BSM-5 were 38.13 W/g, 24.33 W/g, and 20.90 W/g, respectively, representing decreases of 18.16%, 29.83%, and 49.7% compared to the pure BC aerogel. Similarly, the THR exhibited a comparable downward trend, indicating that the introduction of montmorillonite effectively suppressed the thermal decomposition process of the aerogel, thereby significantly improving its thermal stability. The results demonstrate the effectiveness of the BC/SA dual network and MMTs in enhancing the thermal stability of the composite aerogel system [43].

The flame retardancy of BS and BSM aerogels is measured from two aspects: intrinsic flame resistance (LOI) and combustion behavior (UL-94 vertical burning test). Firstly, its intrinsic flame resistance is quantified through the Limiting Oxygen Index (LOI) test (Figure 5f). The LOI value of pure BC aerogel is only 22.3%, indicating that it is significantly flammable. When SA is introduced to construct a BC/SA dual network, the resulting BS aerogel has an LOI value increased to 32.2%, reaching the flame-retardant category. Further research shows that the addition of MMTs has a significant synergistic flame-retardant effect. The LOI value of BSM-1 increases to 41.9%. With an increase in the amount of MMTs added, the LOI value significantly improves: LOI of BSM-3 is 62.6%, LOI of BSM-5 is 69.3%. Secondly, the combustion behavior of BS and BSM aerogels were systematically evaluated through a UL-94 vertical burning test (Table 3 and Figure S12). Pure BC aerogel exhibited poor flame resistance, undergoing rapid combustion upon flame contact, with over 50% mass loss within 5 s. Although no sustained flaming was observed after flame removal, persistent smoldering led to complete consumption of the sample within 70 s, failing to meet any UL-94 classification criteria. The incorporation of SA resulted in measurable improvement in flame retardancy. While still non-compliant with UL-94 standards, the BS aerogel maintained relatively intact structural integrity post-combustion and demonstrated a 25 s reduction in total burning duration compared to the pure BC aerogel. Notably, the BSM-1 aerogel containing 1 wt.% MMTs displayed significantly enhanced flame retardancy, achieving UL-94 V-1 rating with self-extinguishing times of 13 s and 3 s after the application of the first and second flames, respectively, yielding a total combustion duration of 16 s. As the content of MMT increases, the flame retardancy rating of the BSM aerogel (3 wt.% MMTs) significantly improves. BSM-3 aerogel attained UL-94 V-0 classification, with a total burning time of only 6 s. BSM-5 aerogel (5 wt.% MMTs) achieved the optimal performance, demonstrating instantaneous self-extinguishing behavior upon application of both flames while maintaining its V-0 rating. Overall, LOI and the UL-94 test showed the excellent flame retardancy of BS and BSM aerogels, confirm that flame retardancy can be gradually optimized by the BC/SA dual-network and inorganic MMT.

Furthermore, to visually demonstrate the fire protection performance of BSM aerogel as a flame-retardant material in practical applications, a BSM-3 sample (25 mm × 25 mm × 10 mm) was placed on an asbestos mesh for combustion testing (Figure 5g,h). A rabbit-shaped chocolate was positioned on one side of the sample as a combustible indicator, while a butane flame of approximately 1300 °C was applied to the opposite side. Observations revealed that upon contact with the flame, the BSM-3 sample rapidly carbonized but exhibited no flame penetration during the 5 min continuous burning test. The chocolate adjacent to the aerogel surface showed slight melting but retained its overall structural integrity, effectively preventing ignition on the protected side. In contrast, the commercial foam was instantly perforated upon contact with the flame, completely combusting within seconds, with the flame rapidly penetrating the material and igniting the chocolate. These results conclusively demonstrate the exceptional thermal insulation and fire-resistant performance of the BSM aerogel, far surpassing that of traditional commercial foam materials.

### 3.5. Residual Char Analysis and Flame-Retardant Mechanism

**Figure 6:**
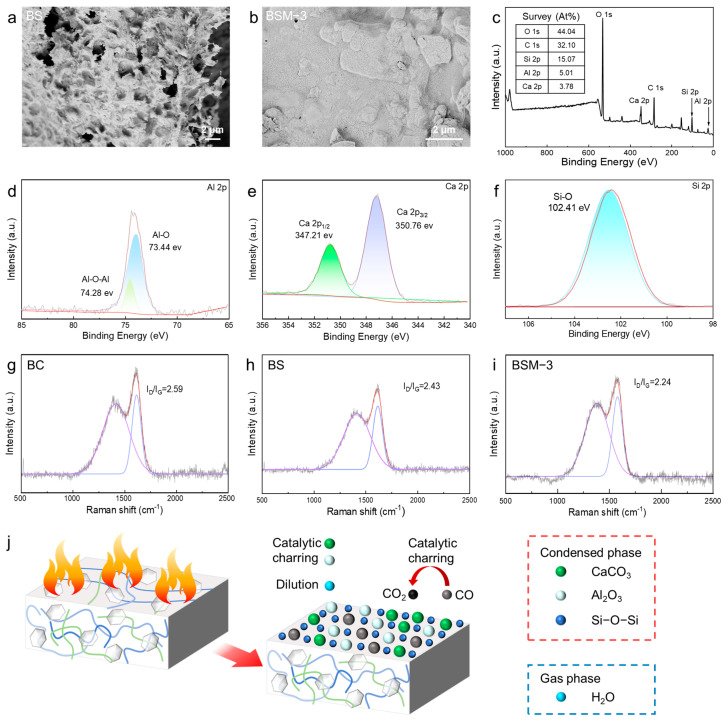
Residual char analysis and flame-retardant mechanism. SEM images of char residues after combustion of aerogel samples: BS (**a**), BSM-3 (**b**); (**c**) survey XPS measurement spectra of BSM-3. High-resolution spectra of Al 2p (**d**), Ca 2p (**e**), and Si 2p (**f**). Raman spectra of char residues: BC (**g**), BS (**h**), and BSM-3 (**i**). Possible flame-retardant mechanisms of BSM aerogel (**j**).

Compared to pure BC aerogel, the flame-retardant performance of BSM has significantly improved, which is speculated to be due to the synergistic effect of MMT and the Ca^2+^ cross-linked network (Network I), catalyzing the formation of a dense char layer. Additionally, the layered structure of the inorganic nanosheets further enhances the barrier effect, thereby improving fire resistance. As SEM images show, pure BS aerogel residue exhibits a characteristic loose, porous architecture with prominent irregular surface protrusions, structural features indicative of inadequate protective layer formation during pyrolysis (Figure 6a). In contrast, BSM-3 aerogel residue demonstrates superior structural densification, manifesting as a continuous char layer containing negligible nanoscale pores while maintaining structural integrity (Figure 6b). This distinctive microstructure plays a pivotal dual role in flame retardation: the dense char matrix effectively hinders flame penetration into the internal substrate, while the nanoscale pores on the continuous char layer are significantly smaller than the mean free path of the gas, thus substantially suppressing the flow of combustible gases and achieving dual protection. This dense, continuous char layer structure is logically consistent with the extremely high LOI value and UL-94 V-0 rating, as it effectively inhibits mass and heat transfer during combustion, providing strong indirect evidence for the condensed-phase flame-retardant mechanism.

To further analyze the mechanism for the formation of dense char layer, high-resolution XPS spectra further elucidate the chemical states of each element. As shown in Figure 6c, the mass fractions of the main elements in the residual carbon are as follows: carbon (C) accounts for 44.04 At%, oxygen (O) for 32.10 At%, silicon (Si) for 15.07 At%, aluminum (Al) for 5.01 At%, and calcium (Ca) for 3.78 At%. In the Ca 2p spectrum, the characteristic peaks at 347.21 eV and 350.76 eV correspond to the 2p_1_/_2_ and 2p_3_/_2_ orbitals of CaCO_3_, confirming the presence of CaCO_3_ in residual carbon [45]. On the Si 2p spectrum, a distinct peak at 102.41 eV is assigned to the Si-O-Si bond, verifying the existence of SiO_2_. In the Al 2p spectrum, the double-peak structure at 73.33 eV and 74.28 eV originates from the Al-O bond and the Al-O-Al structure, indicating that Al_2_O_3_ is generated from the pyrolysis of MMT (Figure 6d–f) [46]. More importantly, the formation of the Al-O-Si structure indicates that the Al^3+^ coordinating at the edges of MMT layers possesses Lewis acidic characteristics. During pyrolysis, this characteristic has the potential to significantly catalyze carbonization. The presence of silicate minerals may also promote the densification of the carbon layer, thereby enhancing the flame retardancy of the composite material. As a proof of concept, the degree of graphitization of the char layers in pure BC, BS, and BSM aerogels were analyzed. Raman spectroscopy reveals that the incorporation of SA and MMT significantly enhanced the graphitization degree of residual char (Figure 6g–i). With the adding of a Ca^2+^ cross-linked network and MMTs, I_D_/I_G_ (the intensity ratio of D-band 1410 cm^−1^ to G-band 1610 cm^−1^) decreases (2.59 for pure BC, 2.43 for BS, 2.24 for BSM-3), indicating an increased proportion of ordered sp^2^ hybridized carbon structures [47]. This structural evolution originated from the synergistic effect of MMT and Ca^2+^ in the SA network in promoting the formation of graphitic microcrystals while suppressing amorphous carbon generation during pyrolysis [44]. The resulting dense graphitized char layer effectively impeded heat and oxygen diffusion through enhanced thermal stability and physical barrier effects, thereby improving the flame-retardant performance of the composite.

Based on the above characterization, the potential flame-retardant mechanisms of the MMT nanosheet and Ca^2+^ cross-linked network can be proposed as follows (Figure 6j): first, MMT is uniformly dispersed within the aerogel matrix, where its two-dimensional lamellar structure acts as a physical barrier to effectively hinder heat transfer [46]. During combustion, MMT gradually decomposes into SiO_2_ and Al_2_O_3_ nanoparticles, while the calcium alginate in the matrix decomposes into CaCO_3_ [43,44,45,48]. Through metal oxide-catalyzed carbonization, residual char grows on their surfaces, promoting the potential formation of a continuous and compact char layer [49]. Simultaneously, the silicon in MMT participates in constructing a silicaceous char layer, possibly enhancing its stability to effectively isolate oxygen and heat penetration while preventing the escape of flammable gases (e.g., CO, CH_4_) and smoke [46]. Moreover, MMT itself exhibits high thermal stability (decomposition temperature > 500 °C), delaying the thermal degradation of the aerogel skeleton and reducing the combustion rate. The undecomposed MMT layers contain abundant hydroxyl (-OH) and siloxane (Si-O-Si) groups on their surfaces, which may physically adsorb or chemically scavenge free radicals (e.g., -OH, -H) in the combustion chain reaction, thereby suppressing gas-phase flaming [48]. Additionally, the H_2_O released during decomposition dilutes flammable gases [47]. Beyond these mechanisms, the inherently low thermal conductivity of BSM aerogel restricts heat propagation, further improving the flame retardancy. These advantages collectively enhance the fire safety of BSM aerogel.

## 4. Conclusions

In summary, an aerogel with a BC/SA dual network, along with an MMT inorganic nanosheet layer structure, has been designed, exhibiting excellent thermal insulation and flame retardancy. The BC/SA network, reinforced by dual cross-linking of GLD and Ca^2+^, enhances the cell wall of aerogel, enabling it to withstand the solvent surface tension during the drying process and maintain the integrity of the microstructure, thereby achieving efficient ambient pressure drying. The introduction of MMT nanosheets endows BSM aerogel with a certain degree of flexibility and lightweight, high-strength characteristics. The incorporation of a BC/SA dual network and MMT significantly improves the flame retardancy of BSM aerogel compared to pure BC. This design strategy, combining a dual-network matrix with inorganic nanosheet layers, allows for the flame-retardant modification of cellulose aerogels while enabling efficient and cost-effective ambient pressure drying, providing a new pathway for the flame-retardant design and efficient fabrication of cellulose aerogels.

## Figures and Tables

**Table 1:** Volume of samples before and after drying and apparent density after drying (V_0_ means volume before drying, V_1_ means volume after drying). The apparent density of the aerogel is calculated by dividing its mass by volume.

Sample	Mass/g	V_0_/cm^3^	V_1_/cm^3^	Shrinkage Ratio	Density g/cm^3^
BC	0.08	10.21	3.88	62.01%	0.021
BS	0.14	10.03	7.26	27.63%	0.019
BSM-1	0.24	10.03	9.14	8.97%	0.027
BSM-3	0.44	10.32	9.70	6.00%	0.045
BSM-5	0.64	10.01	9.61	3.54%	0.066

**Table 2:** Thermal decomposition parameters of aerogel composites in air atmosphere.

Sample	BC	BS	BSM-1	BSM-3	BSM-5
T_5%_ (°C)	69.83	76.83	77.5	77.83	80.17
R_5%_ (%/°C)	−1.24	−1.8	−1.22	−1.42	−1.21
T_max%_ (°C)	319	301	303	297	298
R_max%_ (%/°C)	−6.00	−1.94	−1.08	−0.80	−0.79
Char yield (wt.%)	9.09	32.03	27.31	53.2	59.60

**Table 3:** UL-94 vertical burning test parameters of aerogel composites in air atmosphere.

Samples	LOI (%)	UL-94				
		t_1_ (s) ^a^	t_2_ (s) ^b^	t_1_ + t_2_ (s)	Rating	Melt Dropping
BC	22.3 ± 0.2	60	-	60	N.R	No
BS	32.2 ± 0.3	35	-	35	N.R	No
BSM-1	41.9 ± 0.3	13	3	16	V-1	No
BSM-3	62.6 ± 0.3	4	2	6	V-0	No
BSM-5	69.3 ± 0.5	0	0	0	V-0	No

^a^ t_1_ is the duration of the sample burning after the first flame is applied. ^b^ t_2_ is the duration of the sample burning after the second flame is applied.

## Data Availability

The original contributions presented in this study are included in the article and Supplementary Material. Further inquiries can be directed to the corresponding author(s).

## Supplementary Materials

The following supporting information can be downloaded at: https://www.mdpi.com/article/10.3390/polym17172377/s1, Figure S1. TEM images of MMT nanosheet and BC nanofiber. Figure S2. X-ray Diffraction XRD Analysis of MMT. Figure S3. SEM image of MMT nanosheets. Figure S4. FTIR curves of pure SA and Ca^2+^ cross-linked SA. FTIR image shows that after soaking in Ca^2+^ ions, the characteristic peak of νasym (COO^−^) (~1610 cm^−1^) in SA red shifts into a lower wavenumber, indicating that SA undergoes cross-linking with ions, resulting in the consumption of functional groups. Figure S5. Optical photograph showing the cross-linking of BC/SA by adding GLD. Figure S6. GLD-cross-linked dual-network BS aerogel exhibits excellent durability, remaining intact without decomposition when placed in a flowerpot for 30 days. Figure S7. Schematic of large-scale production. Schematic diagram of efficient mass production by continuous freezing and atmospheric pressure drying. Figure S8. Compressive σ–ε stress-strain curves of BS, BSM-3 and BSM-5 aerogels. Figure S9. σ–ε curves of BS, BSM-3 and BSM-5 aerogels during 20 cycles of compressing fatigue resistance test (ε = 30%). Figure S10. IR photographs of melamine foam and BS aerogels recorded with for 300 s which show surface temperature change of each sample during test. Figure S11. (a) Schematic of the heat transfer process in aerogel (taking BSM-1 as an example). (b) Heat flux density of BS and BSM aerogels demonstrate excellent thermal insulation performance. Figure S12. Screenshots from UL-94 videos of BS and BMS aerogels. Table S1. Total financial cost comparison table (time cost required to dry 1 m^3^ of samples). Table S2. Total time cost comparison table (time cost required to dry 1 m^3^ of samples). References [24,28,29,50,51,52] are cited in the supplementary materials.
