# Supplementary material for: Dual-Network Thermal-Insulating and Flame-Retardant Cellulose Aerogel Fabricated via Ambient Pressure Drying
Source: Polymers (Basel). 2025 Aug 31;17(17):2377. doi: 10.3390/polym17172377 (PMC12431183; doi:10.3390/polym17172377)
Supplement: Supplementary file 1 [file polymers-17-02377-s001.zip › polymers-3829044-supplementary.pdf]

polymers

MDPI

*Article*

# Dual-Network Thermal-Insulating and Flame-Retardant Cellulose Aerogel Fabricated via Ambient Pressure Drying

**Zhengsong Wu [1,†], Yucheng Gao [2,*,†], Shibin Nie [2,*], Dongyue Zhao [1] and Xudong Cheng [3]**

[1] School of Safety Science and Engineering, Anhui University of Science and Technology, Huainan 232001, China; 2023200165@aust.edu.cn (Z.W.); zhaody2022049@aust.edu.cn (D.Z.)
[2] School of Public Security and Emergency Management, Anhui University of Science and Technology, Hefei 231131, China
[3] State Key Laboratory of Fire Science, University of Science and Technology of China, Hefei 230027, China; chengxd@ustc.edu.cn
* Correspondence: gaoyc@mail.ustc.edu.cn (Y.G.); nieshibin88@163.com (S.N.)
† These authors contributed equally to this work.

**Table S1.** Total financial cost comparison table (time cost required to dry 1 $m^3$ of samples).

| | APD | FD |
|---|---|---|
| Electricity cost | CNY 3,225 | CNY 108 |
| Material cost | CNY 1,120 | CNY 3,785 |
| Equipment cost | CNY 266,000 | CNY 4,000 |
| Total financial cost | CNY 270,345 | CNY 7,893 |

**Table S2.** Total time cost comparison Table (time cost required to dry 1 $m^3$ of samples).

| | APD | FD |
|---|---|---|
| Total Batches | 112 batches | 10 batches |
| Daily Production Capacity | 9 L | 70 L |
| Total Time cost | 112 days | 15 days |

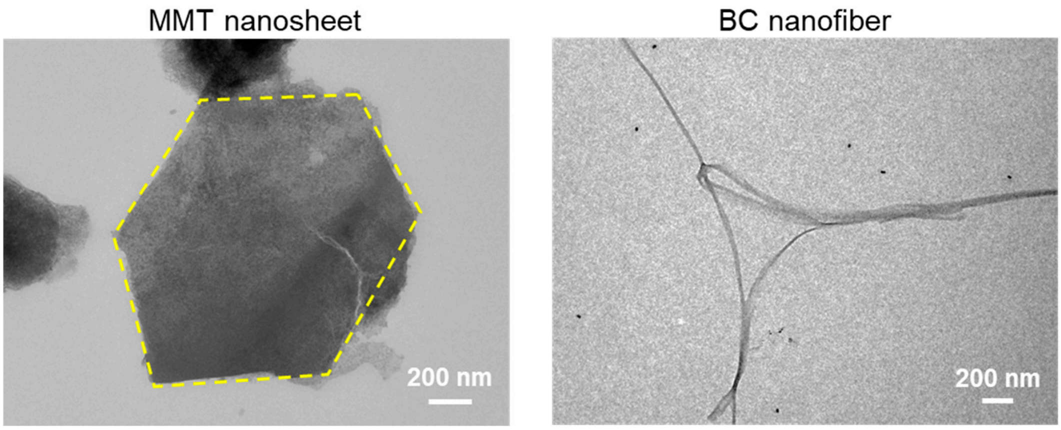


**Figure S1.** TEM images of MMT nanosheet and BC nanofiber.

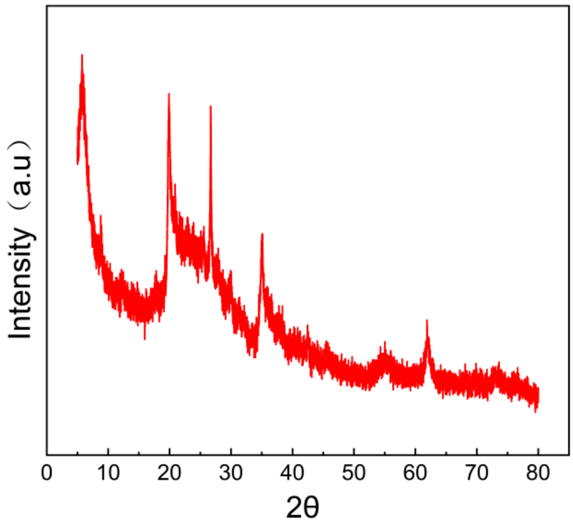


**Figure S2.** X-ray diffraction XRD analysis of MMT.

X-ray diffraction (XRD) is an important method for characterizing the crystal structure of nanomaterials. The XRD pattern of MMT is depicted in Figure S1. A distinct (001) diffraction peak was observed at 2θ = 5.81°[1, 2], and the layer thickness was calculated using the Scherrer equation：

$$D=\frac{K\lambda}{\beta\cos\theta}$$

K: shape factor (typically ranging from 0.89 to 0.94, with 0.9 recommended for flake-like materials) $\lambda$: X-ray wavelength (0.15406 nm for Cu K$\alpha$ radiation); β: corrected full width at half maximum (FWHM) θ: Bragg angle.

Thus, the thickness D was calculated to be 7.98 nm, confirming the successful exfoliation of montmorillonite nanosheets[3].

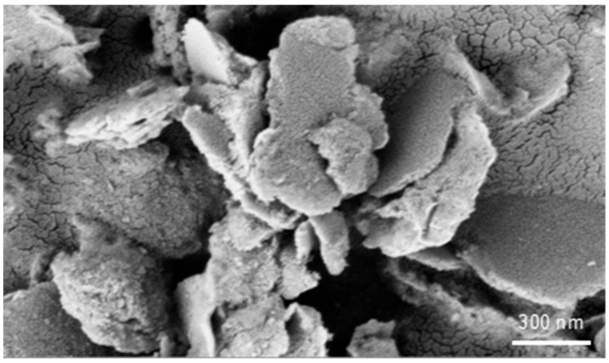


**Figure S3.** SEM image of MMT nanosheets.

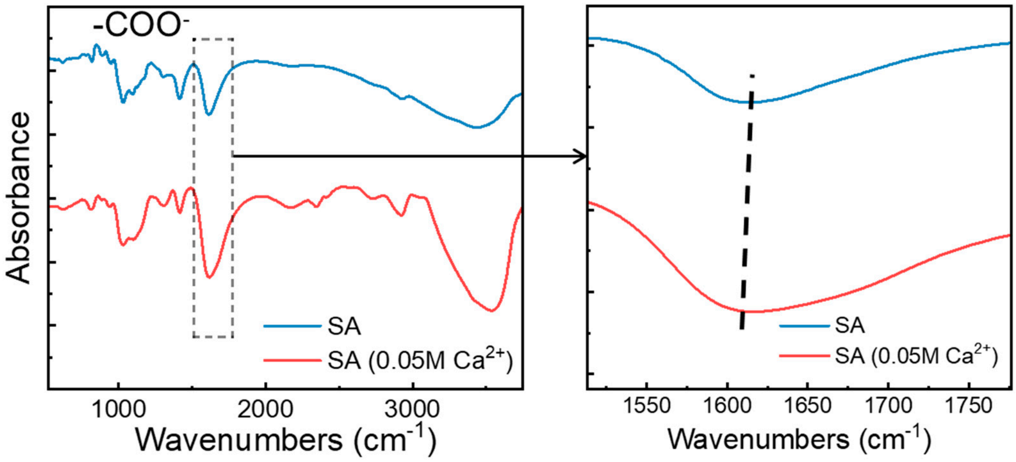


**Figure S4.** FTIR curves of pure SA and $Ca^{2+}$ cross-linked SA. FTIR image shows that after soaking in $Ca^{2+}$ ions, the characteristic peak of $\nu_{asym}$ ($COO^-$) (~1610 $cm^{-1}$) in SA red shifts into a lower wavenumber, indicating that SA undergoes cross-linking with ions, resulting in the consumption of functional groups[4, 5].

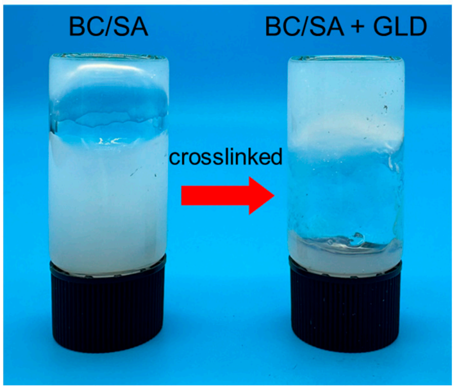


**Figure S5.** Optical photograph showing the cross-linking of BC/SA by adding GLD.

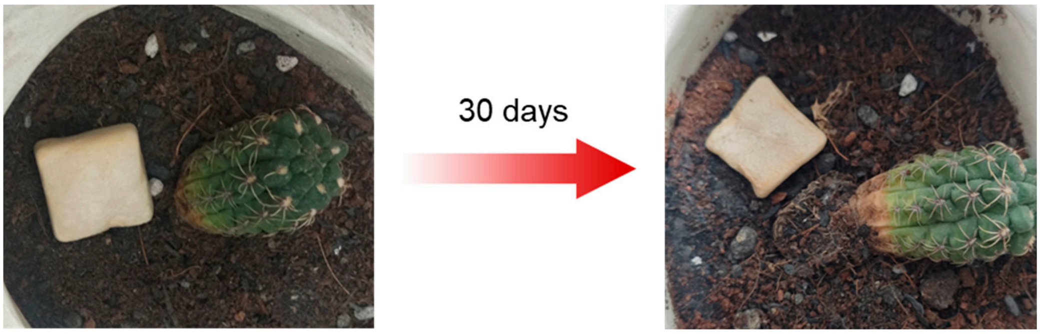


**Figure S6.** GLD cross-linked dual-network BS aerogel exhibits excellent durability, remaining intact without decomposition when placed in a flowerpot for 30 days.

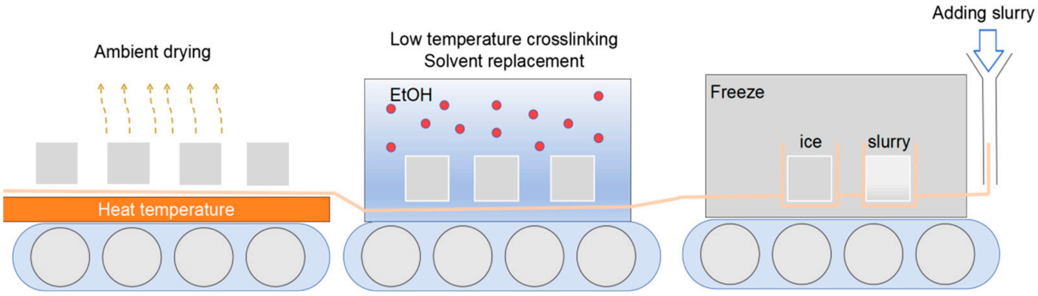

**Figure S7.** Schematic of large-scale production. Schematic diagram of efficient mass production by continuous freezing and atmospheric pressure drying.

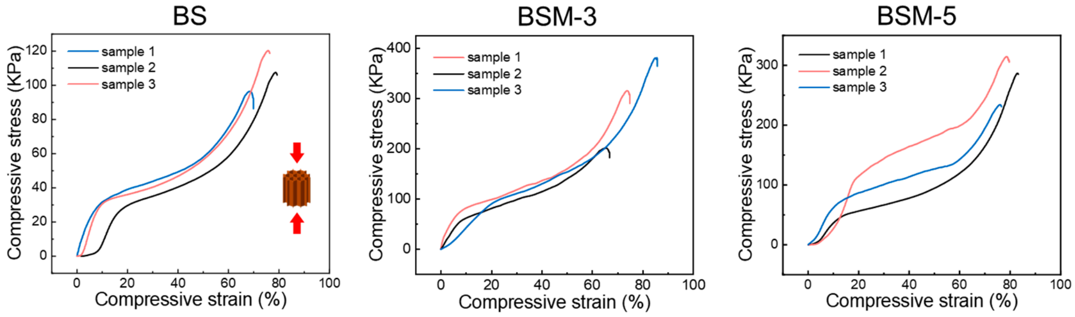


**Figure S8.** Compressive σ–ε stress–strain curves of BS, BSM-3, and BSM-5 aerogels.

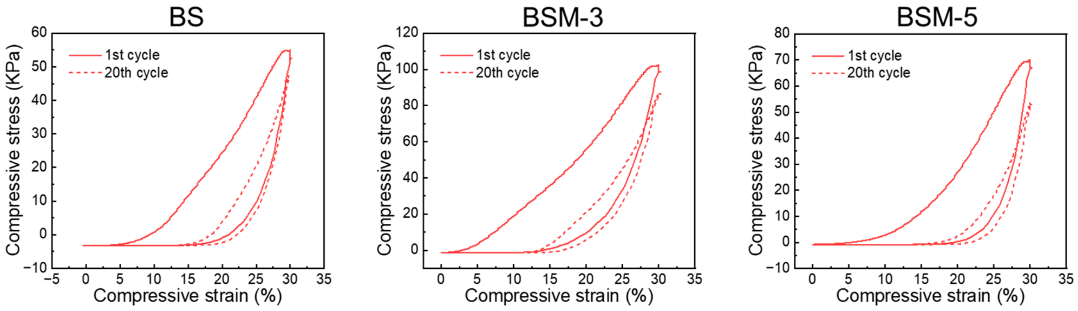


**Figure S9.** σ–ε curves of BS, BSM-3, and BSM-5 aerogels during 20 cycles of compressing fatigue resistance test (ε = 30%).

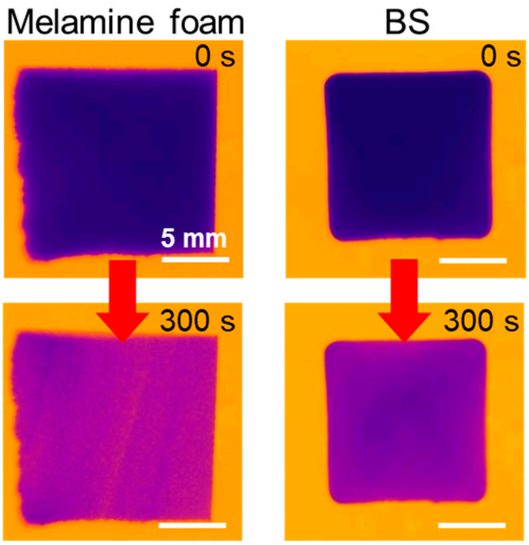


**Figure S10.** IR photographs of melamine foam and BS aerogels recorded for 300 s and showing surface temperature change of each sample during test.

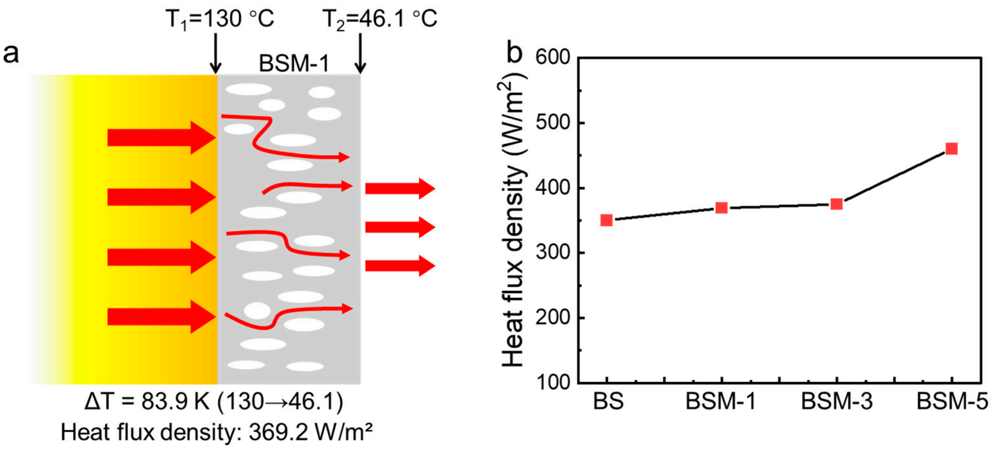


**Figure S11.** (a) Schematic of the heat transfer process in aerogel (using BSM-1 as an example). (b) Heat flux density of BS and BSM aerogels demonstrate excellent thermal insulation performance[6].

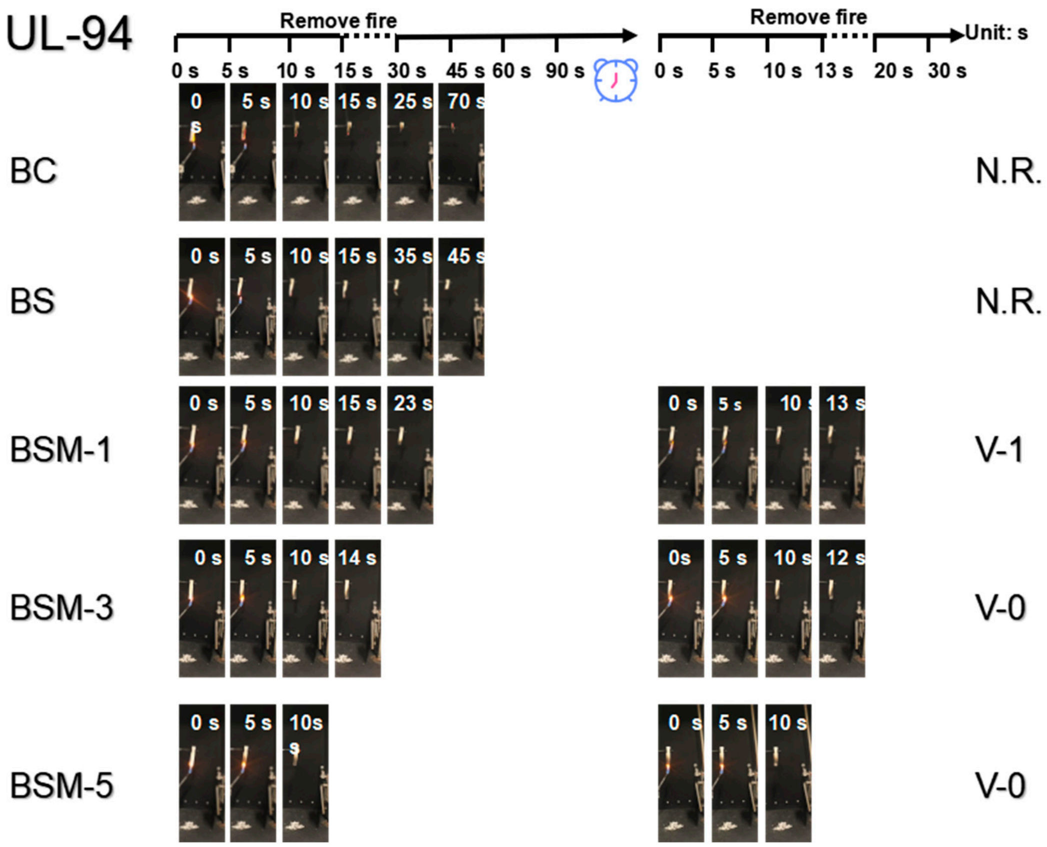


**Figure S12.** Screenshots from UL-94 videos of BS and BMS aerogels.

## References

[1] Q.H. Kong, J.H. Zhang, J.J. Ma, C.W. Yi, F.C. Li, H. Liu, W.L. Lu, Flame Retardant and Smoke Suppressant of Fe-Organophilic Montmorillonite in Polyvinyl Chloride Nanocomposites, Chin. J. Chem. 26(12) (2008) 2278-2284. https://doi.org/10.1002/cjoc.200890404.

[2] E.P. Rebitski, P. Aranda, M. Darder, R. Carraro, E. Ruiz-Hitzky, Intercalation of metformin into montmorillonite, Dalton Trans. 47(9) (2018) 3185-3192. https://doi.org/10.1039/c7dt04197g.

[3] S. Nie, Z. Zhao, W. Zhai, J. Yang, H. Zhang, D. Zhao, J. Wang, Interfacial property optimization through the co-deployment of MOF-derived nickel phyllosilicate and DOPO: Effective reinforcement and flame retardancy of epoxy resin, Composites, Part B 289 (2025) 111947. https://doi.org/10.1016/j.compositesb.2024.111947.

[4] Y.C. Gao, B. Qin, S.M. Wen, Y. You, J. Xue, Y.C. Yin, Z.Y. Ma, K. Dong, Y.F. Meng, I. Manke, S.C. Zhang, Z.L. Yu, S.H. Yu, Ambient Pressure Drying of Freeze-Cast Ceramics from Aqueous Suspension, Nano Lett. 23(19) (2023) 9011-9019. https://doi.org/10.1021/acs.nanolett.3c02654.
[5] C. Han, S.B. Nie, Z.G. Liu, S. Liu, H. Zhang, J.Y. Li, H.R. Zhang, Z.H. Wang, A novel biomass sodium alginate gel foam to inhibit the spontaneous combustion of coal, Fuel 314 (2022). https://doi.org/10.1016/j.fuel.2021.122779.
[6] Z.M. Han, W.B. Sun, K.P. Yang, H.B. Yang, Z.X. Liu, D.H. Li, C.H. Yin, H.C. Liu, Y.X. Zhao, Z.C. Ling, Q.F. Guan, S.H. Yu, An All-Natural Wood-Inspired Aerogel, Angew. Chem., Int. Ed. 62(6) (2023) e202211099. https://doi.org/https://doi.org/10.1002/anie.202211099.
